# Endoscopic submucosal dissection of rectal lesion recurrence at the anastomosis site: when the staples lead the way

**DOI:** 10.1055/a-2058-8321

**Published:** 2023-04-26

**Authors:** Ana Rita Franco, André Mascarenhas, Raquel R. Mendes, Catarina O’Neill, Bruno Pereira, Pedro Barreiro, Cristina Chagas

**Affiliations:** 1 Department of Gastroenterology, Centro Hospitalar Lisboa Ocidental, Portugal; 2 Department of Gastroenterology, Unidade Local de Saúde do Litoral Alentejano, Portugal; 3 Advanced Endoscopy Center, Hospital dos Lusíadas Lisboa, Portugal

A 75-year-old man underwent an anterior rectum resection due to an early neoplastic rectal lesion. On surveillance endoscopy 4 years later, the surgical anastomosis was identified at 8 cm from the anal margin and was associated with a 35-mm lateral spreading tumor (LST), compatible with neoplastic recurrence. The LST reached the anastomosis as well as an adjacent pseudodiverticular recess ( Fig. 1 ). White-light and narrow-band imaging evaluation looking for signs of invasive disease were unremarkable, so endoscopic submucosal dissection (ESD) was decided upon.

**Fig. 1:**
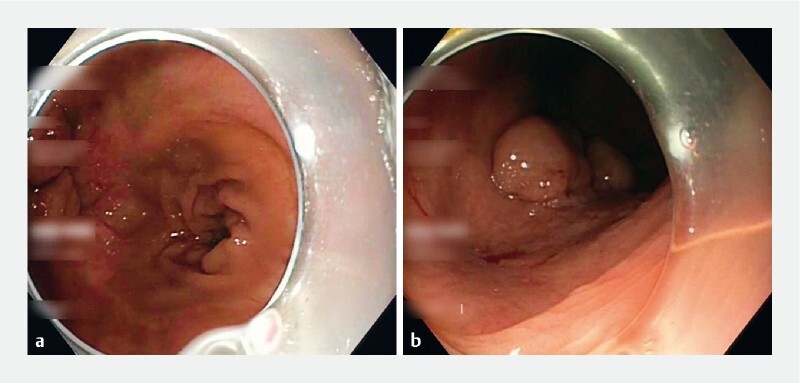
Lateral spreading tumor of the granular type at the anastomosis site 4 years after anterior rectum resection due to an early neoplastic lesion: **a** direct view; **b** inverted view.

The procedure was performed using a Flush Knife BT (1.5 mm; Fujifilm Co., Tokyo, Japan) ( Video 1 ). Submucosal injection of a solution made of polygeline (Gelafundin), indigo carmine, and epinephrine showed the nonlifting sign in the lesion area over the surgical anastomosis. After dissection of the submucosa without fibrosis, an extensive line of 2-cm surgical staples was reached. At this point, the ESD became technically challenging not only due to the presence of the staples, which often hindered passage of the electric current, but also because of the severe associated fibrosis ( Fig. 2 ). During the dissection, two adjacent 2-mm microperforations were noted ( Fig. 3 ); after a failed attempt at closure with through-the-scope clips, these were effectively closed with two over-the-scope clips. En bloc resection was achieved ( Fig. 4 ) and histopathology analysis showed R0 resection of a tubular adenoma with low and focally high grade dysplasia. Six-month endoscopic follow-up showed the ESD scar without any residual lesion.

**Video 1** Endoscopic submucosal dissection of rectal lesion recurrence at the anastomosis site.


**Fig. 2:**
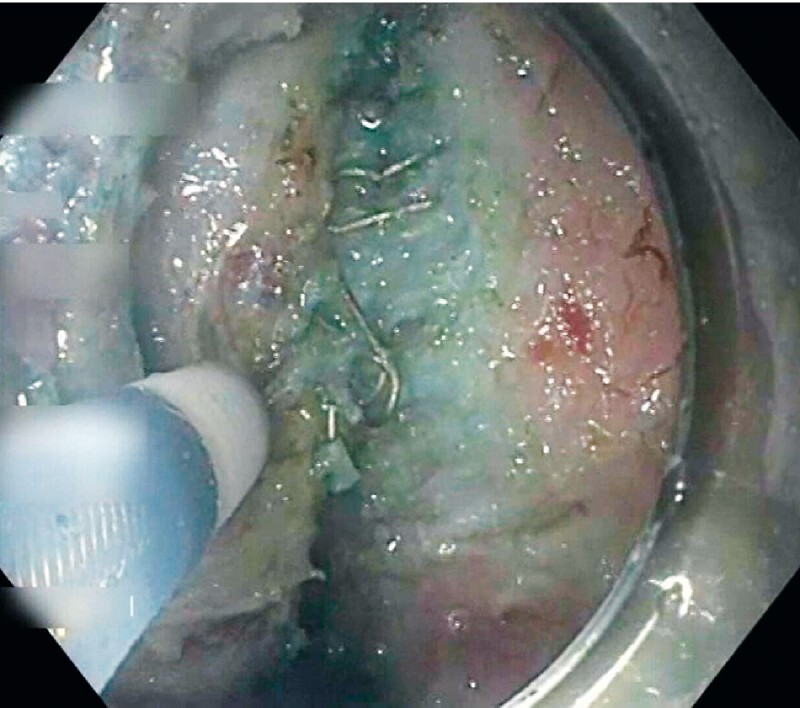
Dissection of submucosa with fibrosis and over the staple line.

**Fig. 3:**
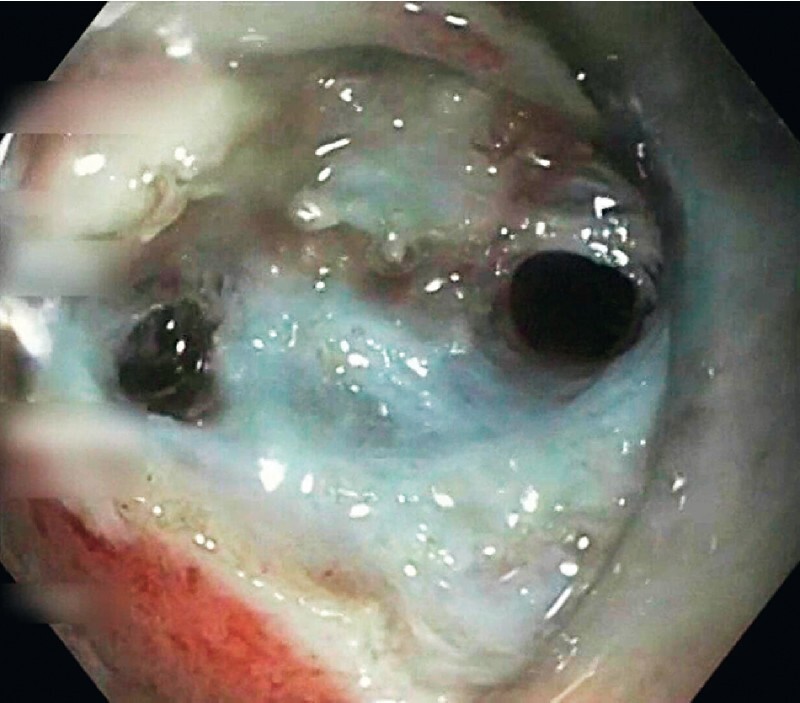
The two adjacent microperforations noted during dissection over the staple line.

**Fig. 4:**
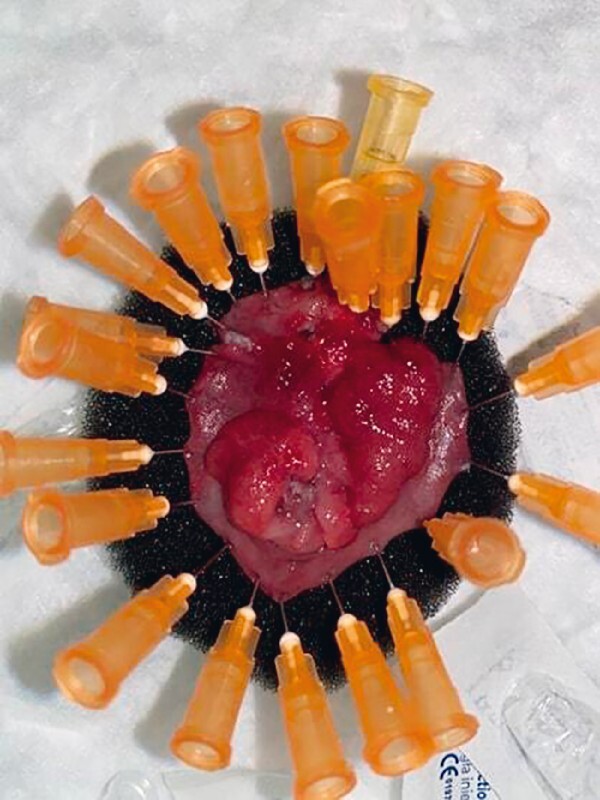
Macroscopic appearance of the resected specimen; dimensions: 45 × 40 mm.

Patients who have undergone colorectal surgery for either cancer or benign lesions continue to be at risk of developing recurrent, residual, or metachronous lesions in the remaining colon, including at the anastomosis site 1 . Surgical resection of a recurrent rectal lesion involves radical en bloc resection of the lesion together with all involved structures followed by major reconstructive procedures; morbidity can be high 2 . ESD is a minimally invasive therapy for superficial neoplastic neoplasms 3 and, in expert hands, is a feasible strategy for lesions at the anastomosis site 1 .

Endoscopy_UCTN_Code_CPL_1AJ_2AD

## Notes

Endoscopy E-Videos

https://eref.thieme.de/e-videos
*E-Videos* is an open access online section of the journal *Endoscopy* , reporting on interesting cases and new techniques in gastroenterological endoscopy. All papers include a high-quality video and are published with a Creative Commons CC-BY license. Endoscopy E-Videos qualify for HINARI discounts and waivers and eligibility is automatically checked during the submission process. We grant 100% waivers to articles whose corresponding authors are based in Group A countries and 50% waivers to those who are based in Group B countries as classified by Research4Life (see: https://www.research4life.org/access/eligibility/ ). This section has its own submission website at https://mc.manuscriptcentral.com/e-videos
